# Significance of acupuncture treatment for medical staff with low back pain: A case report

**DOI:** 10.1002/jgf2.719

**Published:** 2024-07-08

**Authors:** Takuya Masuda, Kenichiro Egawa, Yu Takeshita, Koitchiro Tanaka

**Affiliations:** ^1^ Division of General Internal Medicine & Rheumatology Mitsui Memorial Hospital Chiyoda‐ku Japan; ^2^ Division of Palliative Care Mitsui Memorial Hospital Chiyoda‐ku Japan; ^3^ Department of Traditional Medicine, Faculty of Medicine Toho University Ota‐ku Japan; ^4^ Hokushin‐Kai; Academic Society of Traditional Japanese Acupuncture and Moxibustion Osaka Japan; ^5^ Acupuncture Clinic, Seimei‐in Shibuya‐ku Japan

**Keywords:** acupuncture, low back pain, medical staff, nurse, oriental medicine, presenteeism

## Abstract

A 24‐year‐old female nurse with a 4‐month history of low back pain (LBP) was treated with acupuncture because of difficulty to her working. At the first presentation, the numerical rating scale (NRS) value was 7. After 2 weeks, the NRS value improved to 2–3, and she could bend over better, including when working night shifts. After 4 months, the NRS value remained at 1–2 so her relocating or leaving of absence from the ICU department was avoided. Acupuncture treatment for medical staff with LBP could relieve their pain and improve decreased clinical performance in hospitals or clinics.

## Graphical abstract

This case report describes the case of a medical nurse with low back pain who successfully achieved pain relief and returned to work following acupuncture treatment in a hospital. Acupuncture treatment for medical staff with LBP could relieve their pain and improve declining clinical performance at hospitals or clinics.

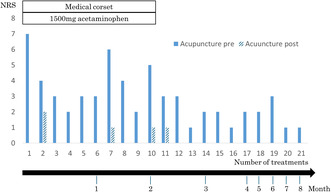

## 1 BACKGROUND

The prevalence of low back pain (LBP) among medical staff, including nurses, is estimated to be 58.7%–75.9%.^1^ Some tend to be unresponsive to painkillers, which restricts or prevents their work and eventually leads to lower‐quality patient care or hospital management.^2^

Recently, acupuncture has been re‐evaluated as a therapy for LBP, with the American College of Physicians' (ACP) guidelines recommending acupuncture for LBP.^3^ However, it is difficult for medical staff to receive this treatment because of poor accessibility. In some cases, acupuncture requires one to three treatment sessions per week, but almost all hospitals or clinics do not provide acupuncture therapy in Japan, or some medical staff (especially nurses) have no time to go to acupuncture clinics due to night‐shift work.

In this case report, we describe the case of a medical nurse with LBP who successfully achieved pain relief and improved declining clinical performance following acupuncture treatment in a hospital.

## 2 CASE PRESENTATION

A 24‐year‐old woman presented with a 4‐month history of severe LBP. She was a nurse of our hospital and suffered LBP after transporting a seriously ill person weighing 100 kg in the intensive care unit (ICU). Lumbar disk herniation was suspected by an orthopedist. However, a lumbar magnetic resonance imaging (MRI) scan showed no evidence of disease, and loxoprofen could not relieve her pain. It was difficult for her to bend over on a bed and work as an ICU nurse, including during night shifts. A diagnosis of myofascial LBP was made, and acupuncture treatment was started.

Her back pain deteriorated with forward and backward bending, and after night‐shift work. She had symptoms of avexing heat in chest, palms, and soles. No fever, numbness, inferior limb weakness, or bladder bowel dysfunction was seen. Upon physical examination, tenderness and tension on the right side of the acupoint *Iyu* (BL21) and flaccidity in *Taikei* (KI3) were seen.

According to an Oriental medical diagnosis (termed “pattern identification” based on ICD‐11), she was categorized as having “liver depression,” “dampness encumbering spleen,” and “kidney deficiency.” It was speculated that “kidney deficiency” due to night shift work dominated the etiology of her LBP.

**FIGURE 1:**
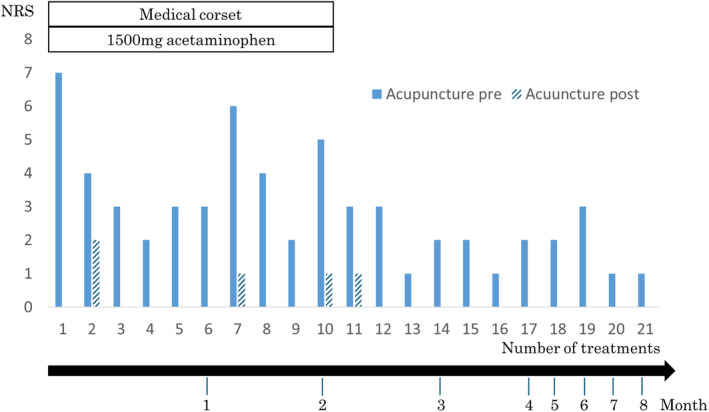
Her pain improved to an NRS value of 0–2 after 10–15 minutes of the each treatment session. NRS, numerical rating scale.

We treated the patient with *Hokushin‐kai*, a traditional Japanese style of acupuncture and moxibustion.^4^ One or two sterilized disposable needles (Seirin Co. Shizuoka, Japan or I'SSIN Co. Hyogo, Japan) were inserted into each acupoint for 10–15 min with no manipulations (depth of 4–10 mm at each acupoint). Each size of needles of diameter 0.2 mm, length 20 mm; diameter 0.18 mm, length 10 mm; and diameter 0.2 mm, length 40 mm was used. Acupoints, needle size, needling method, and retention time of each acupuncture treatment session are shown in Figure 1 and Table 1.

**TABLE 1:** Acupoint, needle size, needling method, and retention time of each acupuncture treatment session. R, right; L, left; D, draining; S, supplementation.

Number of treatments	Acupoint 1	Needle size (mm)	Needling method	Acupoint 2	Needle size (mm)	Needling method	Retention time (min)
1	R, Daiko (ST27)	0.2 × 20	D	L, Syokai (KI6)	0.18 × 10	S	10
2	R, Daiko (ST27)	0.2 × 20	D	R, Kyokuchi (LI11)	0.18 × 10	D	10
3	L, Kokei (SI3)	0.18 × 10	D	‐	‐	‐	10
4	R, Daiko (ST27)	0.2 × 20	D	L, Kyokuchi (LI11)	0.18 × 10	D	10
5	R, Kokei (SI3)	0.18 × 10	D	L, Taikei (KI3)	0.2 × 20	S	10
6	L, Daiko (ST27)	0.2 × 40	S	L, Saninko (SP6)	0.2 × 20	S	15
7	L, Tensei (TE10)	0.2 × 20	S	R, Kyokusen (LR8)	0.2 × 20	D	10
8	R, Daiko (ST27)	0.2 × 20	D	L, Soukai (KI6)	0.18 × 10	S	15
9	L, Syoman (ST20)	0.2 × 20	D	L, Taikei (KI3)	0.2 × 20	S	10
10	L, Kokei (SI3)	0.18 × 10	D	L, Syokai (KI6)	0.18 × 10	S	10
11	R, Suido (ST28)	0.2 × 20	D	R, Saninko (SP6)	0.2 × 20	D	12
12	R, Kokei (SI3)	0.18 × 10	D	L, Taikei (KI3)	0.2 × 20	S	10
13	Cyukan (CV12)	0.2 × 40	D	L, Syokai (KI6)	0.18 × 10	S	10
14	Cyukan (CV12)	0.2 × 40	D	L, Syokai (KI6)	0.18 × 10	S	10
15	L, Kokei (SI3)	0.18 × 10	D	L, Syokai (KI6)	0.18 × 10	S	15
16	L, Kokei (SI3)	0.18 × 10	D	L, Syokai (KI6)	0.18 × 10	S	10
17	L, Katsuniku‐mon (ST24)	0.2 × 20	D	L, Syokai (KI6)	0.18 × 10	S	15
18	L, Syokai (KI6)	0.18 × 10	S	‐	‐	‐	15
19	R, Ashirinkyu (GB41)	0.18 × 10	D	‐	‐	‐	12
20	L, Syokai (KI6)	0.18 × 10	S	‐	‐	‐	10
21	L, Gokoku (LI4)	0.2 × 20	S	L, Syokai (KI6)	0.18 × 10	S	10

**FIGURE 2:**
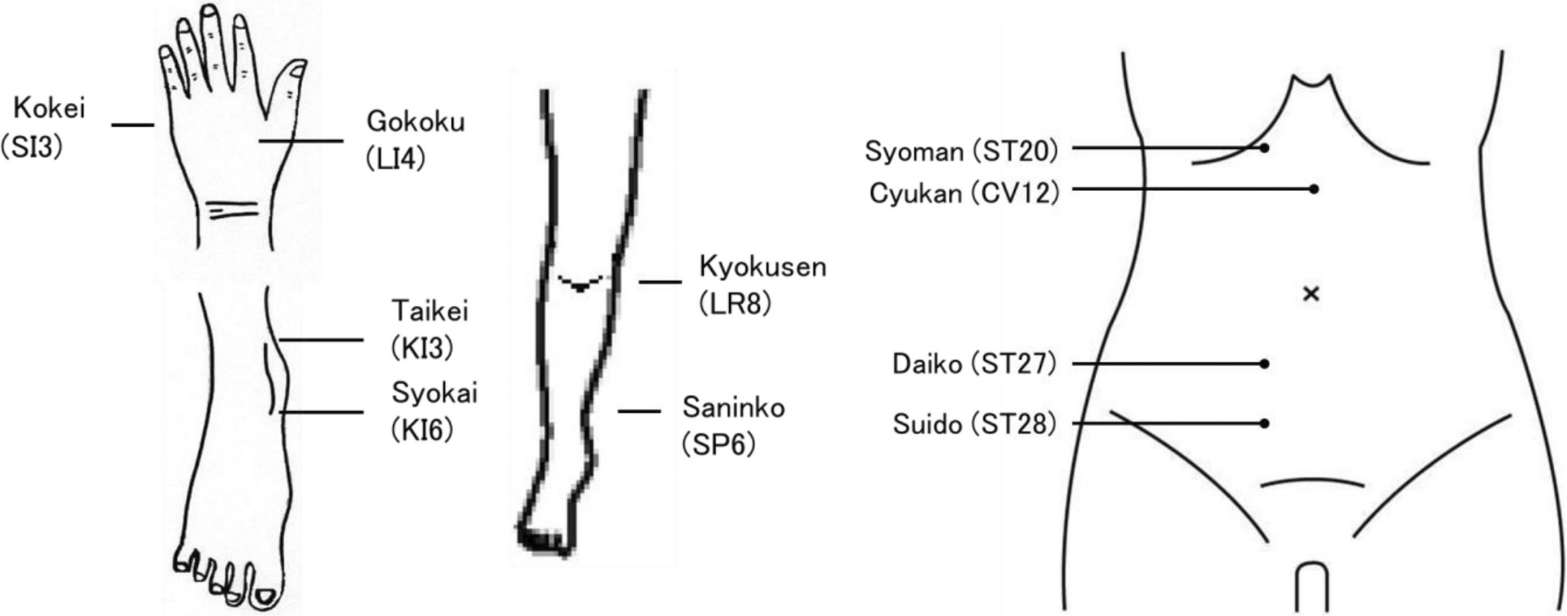
Acupoints for needle insertion were selected with oriental medical diagnosis and acupoint findings such as flaccidity or tension.

At first, the numerical rating scale (NRS) value of her LBP was 7, and there were two treatment sessions per week. Her pain improved to an NRS value of 0–2 after each treatment session. Two weeks later, the NRS value improved to 2–3, and it became easier for her to bend over in her work, including night shifts. Treatment sessions consequently decreased over the following weeks. After 2 months, the NRS value improved to 1–2, and a medical corset or daily administration of 1500 mg acetaminophen was no longer required. After 4 months, the NRS value remained at 1–2, and treatment sessions decreased to once a month (Figure 2). The only symptom that remained was difficulty retaining a seating position for more than 2 hours. No adverse events occurred, and her relocating or leaving of absence from the ICU department was avoided. Acupuncture was performed by a clinical physician, and no cost was incurred to the patient.

## 3 DISCUSSION

Occupational back pain among medical staff, especially nurses, is a serious problem. According to a cross‐sectional study conducted among 254 nurses at Hamad General Hospital in Qatar, the 1‐year prevalence of chronic LBP was 26.8%, 18.1% for sick leave seeking, and 34.3% for medical treatment seeking to LBP.^5^ Another study^6^ revealed that the total costs for 12 months of treatment for nurse's LBP ranged from US$105,405 to 149,083.

Acupuncture first developed around the year 100 BC. Its clinical effect is demonstrated with the insertion of a needle into specific parts of the body termed acupoints. A recent study revealed that the mechanism of pain relief enabled by acupuncture is associated with activating the ascending pain pathway,^7^ even though side effect like malaise (in 7%) was reported.^8^ A previous randomized controlled trial (RCT) showed that laser acupuncture combined with auricular acupressure improved nurses' LBP and quality of life.^9^ Interestingly, a study revealed that financial aid of up to 8000 JPY for acupuncture therapy for workers with symptoms such as LBP may help compensate for losses incurred by organizations in the form of 14,117 JPY per worker per month.^10^ Thus, acupuncture could not only improve declining clinical performance but also improve the profitability of hospitals.

*Hokushin‐kai* style acupuncture^3^ is a traditional form of Japanese acupuncture and moxibustion that we used in this case. One of its characteristics is that only one or two needles are sufficient for each treatment session compared to the 10–20 needles needed for other methods. The price of one needle is around 15 JPY, so *Hokushin‐kai* style acupuncture has excellent value in terms of environmental protection and medical economics.

From an Oriental medical perspective, the clinical presentation of “kidney deficiency” is characterized by tinnitus, aging, decreased libido, nocturia, and LBP. Chronic sleep deprivation may promote “kidney deficiency.” Thus, it is preferable to diagnose night‐shift medical staff with LBP and treat “kidney deficiency” with a Kampo drug (like *Rokumigan*) or acupuncture, even if their LBP is unresponsive to painkillers. *Taikei* (KI3) or *Syokai* (KI6), in particular, is preferred for the treatment of “kidney deficiency.”

Many of medical staffs with LBP are bothered to their pain control which restricts or prevents their work. Acupuncture would be one of the keys to resolve declining their clinical performance as well as their pain. Further investigations are needed to reveal the efficacy of acupuncture to medical staffs with LBP.

## 4 CONCLUSION

Acupuncture treatment for medical staff with LBP might be one of the keys to relieve their pain and improve their declining clinical performance at hospitals or clinics.

### AUTHOR CONTRIBUTIONS

Kenichiro Egawa, Yu Takeshita, and Koitchiro Tanaka revised the text.

### CONFLICT OF INTEREST STATEMENT

The authors declare no conflict of interest.

## ETHICS STATEMENT

Patient consent statement: The patient provided a written informed consent to present the case.

Ethics approval statement: None.

Clinical trial registration: None.
